# A Comprehensive Review of Biochemical Insights and Advanced Packaging Technologies for Shelf-Life Enhancement of Temperate Fruits

**DOI:** 10.3390/bios16020094

**Published:** 2026-02-02

**Authors:** Sharath Kumar Nagaraja, Puneet Kumar, Kavitha R, Sajad Un Nabi, Javid Iqbal Mir, Mahendra Kumar Verma, Ozgun Kalkisim, Mustafa Akbulut, Yong Beom Kwon, Ho-Min Kang, Sheikh Mansoor

**Affiliations:** 1 ICAR-Central Institute of Temperate Horticulture, Srinagar 191132, J&K, India; 2 Department of Horticulture, Faculty of Agriculture, Recep Tayyip Erdoğan University, 53300 Rize/Pazar, Türkiye; 3 Interdisciplinary Program in Smart Agriculture, Kangwon National University, Chuncheon 24341, Republic of Korea; 4 Agricultural and Life Science Research Institute, Kangwon National University, Chuncheon 24341, Republic of Korea

**Keywords:** sensors, quality, storage, shelf life, smart packaging, coating, post-harvest

## Abstract

Temperate fruits, mostly comprising pome, stone fruits, and berries with immense nutritional benefits and a storehouse of various therapeutic phytochemicals, are prone to several physiological disorders immediately after harvest. The etiology, symptom progression, and decay incidence are influenced by pre-harvest and post-harvest factors, causing significant economic loss with respect to both the energy and economics invested. Respiratory end products, ethylene generation, and enzymatic activities interact to influence the metabolic response and associated biochemical variation. Advanced packaging technologies have emerged as innovative solutions to curtail these post-harvest problems. The design and development of novel packaging technologies need to critically understand the respiratory behavior of the fruits and their associated metabolic functions. A desirable polymer or packaging technology should exhibit enhanced barriers to the gases while providing adequate support to the fruit matrix. In addition, it should also fulfill the role of environmental sustainability and the circular economy. The outcome of this review will highlight the importance of proper post-harvest procedure, appropriate pretreatment, packaging matrix selection, and the storage conditions for effective and enhanced shelf-life storage. Therefore, this review was structured in two phases; the first phase discusses the biochemical understanding of the fruit during storage and transit in response to stress factors. The next phase highlights the various packaging interventions (polymers, biodegradable films, edible coatings, smart packaging, nano-packaging) taken to address these issues, with a key focus on shelf-life enhancement. Further, the key limitations of each technology are appraised.

## 1. Introduction

In the era of increasing risk of chronic disease and lifestyle-associated disorders, protective foods such as fruits and vegetables have occupied a significant portion in the human diet. In support, the World Health Organization (WHO) even suggests a dietary intake of more than 400 g of fruits and vegetables per day to accomplish public nutrition and health targets [1]. Temperate fruits in this category are bestowed with abundant nutritional and nutraceutical properties, with importance established towards ensuring farmers’ livelihood and rural employment. The richer and more attractive fruit chromacity is the result of key, therapeutically important metabolites. The fruit production industry in temperate regions is significantly influenced by climatic patterns, with positivity of diurnal temperature changes, chilling hours, and sufficient sunlight all together having caused the global temperate regions to produce higher quality fruits [2,3]. The morpho-physiological response towards the suggested climate effects is driven by genotype, edaphic factors, dormancy behavior, flowering response, and pollination attributes [4,5,6]. The consumer demand for fresh fruit consumption is continuously increasing, because of which there is a rapid expansion drive in the area and production of these temperate fruits [7].

Temperate fruits are technically grouped as pome (apple, pear, quince), stone fruits (apricot, cherries, plum, peach, nectarine), berries, and nuts (almonds, walnuts, pecan nut, hazelnut), with greater diversity prevalent in growth, production, and processing strategies. While the latter nuts have a reasonable shelf-life storage, the former two categories are highly perishable, which requires critical post-harvest attention. These fruits’ maximum growth, being climacteric, produces an increased ethylene response on exposure to external environments that aggravates the progression of ripening and senescence [8]. This has an impact on the organoleptic properties and causes a variation in the nutritional and phytochemical composition of fruits. In continuation of this cause, the possibility of post-harvest and storage-induced pathogen attacks is increasingly rising [9], thus posing a serious impact on food safety management. Scientific understanding of various post-harvest events occurring in fruits is needed for tailoring the benefits of increased shelf life and nutrient retention of fruits. 

**Figure 1:**
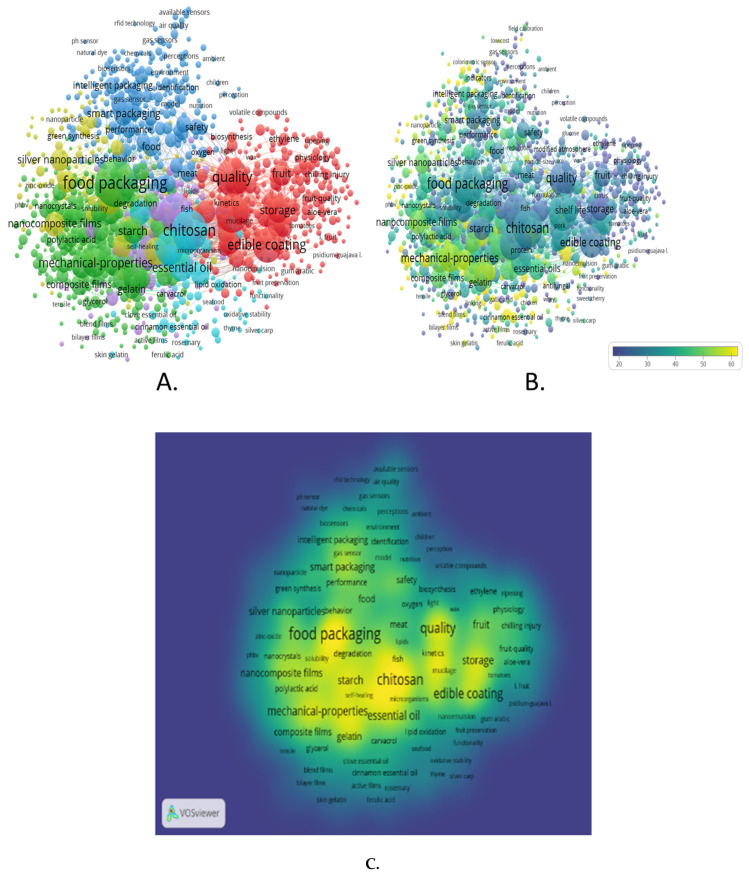
Analysis of keywords related to advances in food packaging: (**A**) displays the average citations of keywords identified by VOSviewer, illustrating the relative importance of specific concepts within the research field. (**B**) Shows keyword co-occurrence, revealing relationships, and clusters of research topics (**C**) provides a density visualization of keywords, highlighting the concentration and distribution of research efforts.

Post-harvest handling of temperate fruits is a critical determinant of quality, safety, and marketability. Given the rapid physiological decay response of the fruits, coupled with the consumer requirement of fruits in their nearly fresh format, the role of packaging has been highly appraised. Packaging serves to deliver the additive of protection and containment, besides being a silent advertiser [10]. In this review, VOSviewer 1.6.20 was employed to explore the interconnections among key themes such as packaging innovation, shelf-life enhancement, and smart packaging technologies. Through bibliometric analysis, the tool highlighted the progression of topics like the integration of sensor technologies and their critical role in maintaining fruit quality during storage and transportation. Furthermore, VOSviewer identified influential authors, journals, and institutions driving advancements in this field, revealing collaborative opportunities and existing knowledge gaps. These analyses provide valuable insights into the broader implications of packaging strategies on post-harvest management, offering guidance for future research aimed at developing sustainable and efficient solutions for temperate fruit preservation (Figure 1).

Packaging innovations in perishable commodities such as fruits should address the respiratory behavior of fruits, in addition to their surface properties, and design a suitable barrier so that the corresponding negative events are withheld. An inverse relationship exists between the respiratory behavior of fruits and their optimal shelf life. The post-harvest shelf life is aimed at creating a microenvironment and providing a barrier from the surrounding atmosphere. In the present context, acute post-harvest loss in freshly harvested fruits poses a significant concern and demands immediate attention. The area has been critically investigated and has driven the development of various packaging technologies such as modified atmosphere packaging, biodegradable polymers, edible coatings, metal oxide-coated multilayered films, active packaging, intelligent packaging, and nanocomposite films. The technologies adapted not only cater to the product requirements, but also meet the demands of eco-friendliness and consumer safety. Newer technologies in polymer film fabrication and better understanding of the product matrix are key deliverables for increased research progress in post-harvest packaging. Despite significant improvements in the packaging arena, there are hardly any comprehensive reviews to be found regarding the impact of advanced packaging technologies on shelf-life extension of perishable commodities with a focus on temperate fruits. Therefore, this review was undertaken in two phases. The first phase critically addresses the cause of various biochemical responses in fruits’ post-harvest stage and continues to the second phase which provides comprehensive research insights carried out so far on the effects of several packaging technologies (basic polymers to advanced packaging) in extending the storage shelf life of fruits.

## 2. Maturity Index and Harvest of Temperate Fruits

Optimum storage stability of fruits depends on proper harvest which in turn depends on the maturity index detection (MI) and harvest initiation. Allowing the fruits to attain maturity ensures satisfactory sensorial appeal due to accumulation of desirable flavor molecules and attainment of appreciable fruit size. The determination of the harvest index can be used to predict the orchard ripening uniformity, growth regulators application, and shelf-life estimation [11]. Commonly used physical/visual methods for harvest determination include fruit size, shape, weight, color, moisture, firmness, soluble solids, titratable acidity, ethylene concentration, chlorophyll degradation, and starch conversion [12,13]. While a few parameters are non-destructive and can be measured at field level, most parameters are subjective and require a laboratory set up for performing the analytical procedures [14]. The post-harvest storage and continuity is being drastically impacted by a minor deviation in harvest mismatch.

**Figure 2:**
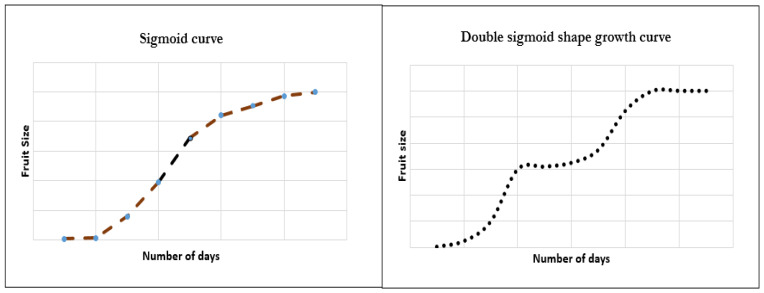
Growth curves of pome and stone fruits.

In the case of apples, following blossom set and pollination, a series of cellular events proceeds, ultimately ending in fruit production. Key molecular events responsible for reaching desirable fruit growth include cell division, cell enlargement, followed by cell differentiation [15]. Pome fruits such as apples are known to follow a sigmoid-type fruit growth (Figure 2) with slower cell division at initial stages, followed by rapid cell expansion at later stages, leading to seed development and peel color change and hardening [16]. The process of fruit set is supported by a continuous flow of photosynthates backed by carbon flow. Often fruits are judged by the moisture attainment, but Vieira et al. [17] established that it is the relative change in total soluble solids (TSSs), starch index, and flesh firmness that becomes a critical harvest indicator and not the dry matter. Any disturbance in the external environment, including soil nutrient or moisture levels, can significantly affect the onset of maturity index. With a higher interest in sensory properties, acidity and soluble solids are essentially critical factors for apples and pears. An inverse relationship between increased sugar is found to correlate with reduction in acidity and firmness. Other methods for harvest prediction include the calculation of days after full bloom (DAFB).

Plums undergo color transition from green to dark red on attaining maturity, which has an indirect role in promoting flavor release and flesh softening [18]. Stone fruits are known to follow a double sigmoid type of fruit set (Figure 2) where two sets of rapid cell growth are caused by a relatively slower cell growth stage [19]. Most stone fruits are non-destructively assayed with the chlorophyll absorbance index (IAD), which is known to exhibit a slower degradation and correlates well with ripeness [20]. Advancements in non-destructive methods for maturity assessment have been the subject of numerous research studies and have yielded a set of interesting methodologies that can be used in real-time and for on-site ripening detection. Fluorescence emission signals were used for maturity assessment in yellow peaches, pomegranates, in which ready-to-harvest fruits had a lower fluorescence intensity (450–650 nm), which is slightly lower as compared with immature fruits that exhibit a greater signal emission (>650 nm) owing to chlorophyll intensity. Generally, color measurements, spectral checks, and imaging studies are being conducted to assess the maturity index [21,22,23].

## 3. Post-Harvest Biochemical Changes in Temperate Fruits

The transformation of physiological state to initiation of biochemical changes in the fruits starts right from maturity and continues till the end of the fruit shelf life. Determining factors of respiration, transpiration, nutrient flux, chlorophyll breakdown, enzyme activity, accumulation of sugars, cell wall component formation, nutritional components, phytochemical composition, and stability are the broader means of visualizing the changes leading to senescence. Quantification and establishing standards at each stage provides a valuable solution to post-harvest problems and can significantly reduce the wastage occurring during the supply chain. Many of the reported studies highlight the correlation between improper harvest and the development of physiological disorders. Various physiological changes and biochemical responses of pome and stone fruits are discussed.

### 3.1. Apples

Apples are known to show a climacteric behavior controlled by two sets of ethylene-regulating systems (I and II). The on-tree initiation of ripening and associated events is purely a varietal character. Varietal influence on storage post-harvest behavior was examined by Giné-Bordonaba et al. [24], where they observed the dependency of Golden Smith apples towards cold storage that could trigger the ethylene release in comparison with the Early Red One variety that produces at 20 °C. Various physiological disorders occur on varying the biochemical activities of the fruits, resulting in damage both externally and internally.

(a) Water loss

Various pre-harvest and post-harvest factors are known to initiate moisture loss leading to shriveling, causing weight loss, and ultimately affecting quality indices in apples [25]. The weight loss can impact the marketability of apples by affecting their packing ratio and specific gravity. Ease of water loss is directly an influencer of excessive internal air space within the fruit. Progressive water loss continues to dry, decay, and soften, ultimately creating favorable conditions for microbial infection. The weight loss can be cultivar- and composition-dependent and the nature of cuticular wax and cell wall thickness [26]. Delicate and fleshy fruit tissue is highly prone to surface damage due to mechanical injury; thereby, increasing the permeability matrix for loss of internal moisture. It is reported that water loss is the primary response to any stress faced by fruit and continues with browning development.

(b) Sunscald

Photo-oxidative damage occurs in excessively radiation-exposed apples, leading to a cascading set of oxidative enzyme release resulting in sunscald symptoms. which is also referred to as delayed sunburn if scalded samples are exposed to lowered temperatures. Sunscald represents one of the major economic loss factors in the apple market which presents as dark, irregular, brown patches. Higher sunlight exposure causes heat stress in the tissues and results in chlorophyll breakdown, photosynthetic protein degradation, tissue leaching, and ending with reduced photosynthetic efficiency. An over-normal amount of sunlight can potentially result in the release of antioxidative enzymes, particularly catalase (CAT), superoxide dismutase (SOD), ascorbate peroxidase (APx), glutathione reductase, dehydroascorbate reductase (DHAR), and monodehydroascorbate reductase [27,28] led by activation of defensive gene transcription.

The protectant molecules, such as oxidized ascorbic acid (dehydroascorbic acid), were found to diminish upon experiencing such heat-stress-induced molecular events. The events also alter the carbohydrate metabolism wherein sugars are transformed into glucose and sorbitol, acting as a major osmotic adjustment in response to sunscald symptoms. This is also supported by the loss of relative water content; thereby, increasing the toughness of the scalded surface [29]. In apples, there was a pronounced increase in xanthophylls at the expense of reduced chlorophyll [28,30] and an increased presence of phytohormones, especially abscisic acid, jasmonic acid, and salicylic acid [30]. Zupan et al. [31] correlated the fact that excessively sunlight-exposed fruit had greater concentrations of peroxidase and total phenolics, which would also have played an additive effect in increasing the scald symptom presentation. Particularly in the case of phenolics, higher concentrations of quercetin and quercetin glycosides had a greater scald development due to their ease of being oxidized and forming brown-colored monomers.

(c) Superficial Scald

Post cold-storage effects of apple results often manifest as superficial scald which is a major cause of economic concern in cold-stored apples. This can be viewed as an advanced stage of chilling injury [32]. The formation of CTs generally occurs in 6–8 weeks in ambient temperatures. These oxidized end products, over a period, accumulate in the form of necrotic hypodermal cells.

(d) Internal Browning

Damage to the static cell compartmentalization leads to the release of phenolics and enzymes, triggering browning reactions resulting in internal browning symptoms. The disorder development is directly linked to pre-harvest factors such as fruit size, fruit weight, nutrient management, adequate irrigation, etc. Broadly, the area of internal browning seems a regular disorder, but the causal factor has the role of chilling injury, senescent-related breakdown (mealy breakdown), and CO_2_ injury [33].

Diffused flesh browning: Chilling injury results when harvested fruits are immediately exposed to lower temperatures making the surface cell membranes vulnerable to undergoing phase separation on account of reduced mobility. This sudden phase change in the membrane drives the way for cellular leakage [34]. The cortex and peripheral tissues are disturbed instantly as they are among the first tissue spaces to witness a sudden change in lower temperatures. Key symptoms include the development of pitting, browning, and eventual decay. The progression is supported by the transcriptome expression studies of over-production in reactive oxygen species (ROS) on encountering lower temperatures and reduced oxygen environments that cannot be counterfeited by host defense systems [35]. This is continued with the release of hydrolytic and lipolytic enzymes which permanently damage the membrane integrity.

Mealy breakdown: Mealy breakdown occurs due to changes in the cellular space, leading to cell leakage. This leakage acts as a greater prerequisite for browning reactions. The word mealy has been attributed to the associated textural damage that occurs and provides a negative addition to the sensory attributes. Another hypothesis suggests the toxic level accumulation of internal CO_2_ might be the factor for cell breakdown.

CO_2_-induced browning: Restriction of gaseous exchange (particularly O_2_) in apples, leading to gradient formation at the core and cortex, leads to the development of a hypoxic environment, continuing with cell death and leakage of metabolites. This is of concern in cultivars that are extremely sensitive to CO_2_ during controlled atmosphere (CA) storage. An excessive leakage of metabolites is immediately associated with the decline in the host defense system and start of oxidative stress. The excessive buildup of stress factors makes the membrane leakage easier. It is observed that the geometrical arrangement and orientation of vascular fruit cells in combination with tissue variability are extremely important factors in building up the gaseous gradient [36]. Another hypothesis suggests the role of ascorbic acid oxidation in CA storage-induced browning [37].

(e) Enzymatic browning:

Enzymatic browning is a serious disorder in sliced or minimally processed apples because of enzyme–phenol interaction leading to the release of brown pigments (melanin). Close to 50% of the stored apples are lost due to its occurrence which not only reduces the sensory appeal but also compromises the nutritional quality [38]. The reason could be more apt for the loss of the host antioxidant defense system and allied components (vitamin C). The presence of polyphenol oxidase (PPO), peroxidase, and the phenolic proportion are key contributors to the browning reaction. Maturity of the fruit is a key indicator for browning, as less mature fruits are prone to lower color difference (ΔE).

The affinity, reactivity, and substrate selectivity of the enzymes are prime factors for browning acceleration. In addition, the presence of various transition metals is known to facilitate the browning process. Substrate dependency behavior of PPO was studied by Marrufo-Hernández et al. [39] where Golden Delicious PPO showed the enhanced oxidation of 4-methyl catechol substrate as compared with ferulic acid. Besides the pre-harvest factors such as irrigation, soil moisture and climate conditions are also related to browning disorder [40]. Phenolic monomers such as catechin, p-coumaric acid, and chlorogenic acid are critical accelerators of PPO, while polymerized proanthocyanins are potential inhibitors of browning. An insight into the possibility of the existence of alternate oxidation pathways was provided by Serra et al. [41], where the team suggested the possible role of indole acetic acid oxidase as the probable cause of browning in varieties of Topaz and Jonathan.

(f) Water core:

A physiological disorder with translucent, watery, intercellular core space accumulation of sugars, polyols because of a deviation in carbohydrate metabolism [42]. The reason is the excessive accumulation of synthesized sorbitol in fruit tissues that has an osmoregulatory effect in attracting water and increasing the hydrophilicity. Excessive water expels air content, leading to a fermentative pathway ending in ethanol and acetaldehyde formation. Prolonged accumulation of sorbitol sap leads to rotting issues with off-flavor development.

### 3.2. Pear

Pear is the second most important pome fruit, abundant in vitamin C, phenolics, and antioxidants; these fruits come with a balanced blend of acidity and sugars [43]. Unlike apples, pears contain polyols in the sugar composition in addition to simple sugars. Immediately after harvest, pear fruits are exposed to the external environment; thereby, an imbalance in the transpiration and respiration profile of fruits is noticed. Too early or too late harvest can accompany associated defects such as improper color development and softening disorder [44]. Changes in fruit flesh are accompanied by biochemical transformation of starch and organic acids, with corresponding changes in firmness, skin color, and sensory profile.

(a) Cork spot

Cork spot is a physiological disorder of complex etiology and is a cause for major market rejection for Asian Pears [45]. The affected fruit presents a round sunken area with lignified flesh beneath the affected skin. It is linked to variations in the functioning of vascular bundles, leading to desiccation and suberization [46]. The effect continues to result in a varied texture due to porosity buildup. Occasionally, scattered areas of necrotic lesions develop in the flesh. Though research has evidenced the imbalance in minerals as the responsible factor for cork spot occurrence, an associated output in the increased levels of antioxidant enzymes is noticed, which suggests the possible involvement of stress factors. The metabolite profiling of cork-spot-affected fruits reveals the presence of lipid, organic acids, and terpenoid metabolites that would have been released from the integrated cell matrix [47].

(b) Internal flesh browning:

An imbalance in gaseous concentration during CA storage leads to the release of enzymes contributing to the browning and decay of pulp tissues. Browning symptoms can be seen as radial arrays, asymmetrical brown spots, dry spots, random brown cavities, and brown core with accumulation of necrotic hypodermal cells [48,49,50,51]. Though all these terminologies are often used interchangeably, there exists a metabolic and biochemical difference post the initiation of each browning symptom. The necrotic spots slowly increase in size and start from the cortex, progressing towards the core. The release of PPO and POD oxidizes phenols with polymerization, producing melanin. In many of the research investigations, polymeric procyanidin has been found to be a major browning agent. Research studies have observed possible cellular blockages because of the shift into fermentative pathways with the accumulation of various end products. The occurrence of hydrogen peroxide radicals can also be a factor for lipid peroxidation and protein denaturation [52,53,54,55]. Lin et al. [56] showed that an increase in ROS is a major factor for browning induction. A pH-dependent occurrence of enzyme tyrosinases and non-latent tyrosinase is observed on the shift in respiration to fermentation mode. In an expanded research investigation, the authors observed an increase in the levels of γ-aminobutyric acid (GABA) and fumaric acid, both of which are known to increase in anoxic conditions [57]. Veltman and Peppelenbos [58] proposed a deficiency in cellular energy as the major reason for browning occurrence.

(c) Superficial scald:

Scald in pears appears as brown to black patches on the external fruit surface that aggravate and manifest themselves into rugose spotting or lenticel spots. The etiology draws from various factors, of which cultivars are the major ones along with maturity at harvest and storage conditions. The initial brown spots cause severe hypodermal necrosis and lead to greater brown to black blotches. During storage, various aroma volatiles start to develop and amongst them is α-farnesene which initially develops on the waxy layer. It is believed that α-farnesene release is greatly correlated with internal ethylene concentration like apples. In contrast to this, Larrigaudière et al. [59] observed a non-dependency of ethylene towards α-farnesene synthesis in winter pear cv. Packham’s Triumph. An interesting output in the investigation was that scald prevalence is no longer a major cause of α-farnesene presence, rather, it is the compromised antioxidant defense system in certain cultivars that shows the sensitivity towards the oxidative conjugated trienes. Oxidation of α-farnesene leads to release of CTs, though they are believed to be non-enzymatic, whereas autoxidation leads to hydro- and endoperoxides (3E,5E and 3E,5Z isomers of farnesyl 7-hydroperoxide, and isomers of farnesyl endoperoxyhydroperoxide) [60]. Together, these end products form CTs and are the major causal metabolite for scald. Excessive accumulation of these CTs interferes with gaseous exchange, resulting in the accumulation of fermentative products. Niu et al. [61] undertook the study on the concentration of various metabolites in response to an increase in scald symptoms. The author found an increase in phenol and antioxidant enzymes (PPO, POD, SOD, CAT). As the concentration of CTs outweighed the antioxidant enzymes, the oxidation of polyphenols and ascorbic acid was accelerated and a corresponding decrease in antioxidant enzymes was observed. In continuation of this, the increased concentration of free radicals initiated the lipid membrane disintegration.

(d) Softening

Excess ethylene production alters the respiratory behavior and accelerates the enzyme release for cell wall degradation, leading to softening [62]. As a result, there is a redistribution of water in the flesh tissues and accumulation of osmolyte occurs, leading to a spongy texture. There is an evident decline in fruit firmness which ultimately is damaged and fetches a lower market price. Delay in harvest coupled with ambient storage conditions accelerates the oxidation reactions and contributes to metabolite breakdown reactions. Depolymerization of pectins leading to an imbalance in cell adhesion causes the leakage and softening effects [63].

### 3.3. Apricot, Peaches, and Nectarine

(a) Brown rot

With this disorder, though caused by fungal growth (*Monilinia* sp.), the pathway in apricots for the pathogen’s entry is made by an imbalance in the physiology of harvested fruit leading to cell wall wounds. During the later phase of maturity, where increased cell expansion occurs, there is a reduction in acidic molecules, increase in sugar compounds, and weakening of the cuticle. All these events allow the quiescent pathogen to become active and initiate the sporulation [64]. The cause may be attributed to the pathogen and the exposed environment. Already, the environment has an increasing respiratory behavior and a lower turgor pressure, and the pathogen growth further accelerates and declines the complete metabolic activity of fruits, causing cell death.

(b) Softening

Enhanced respiratory activity of fruits combined with the ethylene release pattern and cell wall breakdown makes softening a potential threat for long-term storage of apricots and plums. The cause of the event can be related to cultivar type, harvest maturity, and storage conditions [65,66]. Ethylene causes the swelling of cell walls, followed by dissolution by the action of cell wall-degrading enzymes. The pectin fraction is completely solubilized and degrades. Studies have reported that pectin degradation can immediately lead to loosening of pectin cohesion matrix and proceeds to cell separation [67,68]. The firmness of fruit becomes completely lost and is prone to various pathogenic infections.

(c) Gel breakdown:

Gel breakdown in apricots occurs because of chilling injury, where the fruit mass is converted to a translucent mealy pulp, leading to a change in fruit tissue texture and consistency. The symptom presents as gel that is formed adjacent to the stone region and eventually is dispersed to the entire pulp tissue leading to loss of firmness [69]. In apricots, when exposed to a longer duration of cold storage, this leads to an imbalance between oxygen and oxidation products (ROS), thus destabilizing the membrane by toxic radicals. Increase in oxidative damage results in membrane peroxidation and ultimately senescence [20,70].

(d) Pit burn:

A brown discoloration with softening is observed when near-harvest fruits are exposed to higher temperatures. It is also referred to as heat injury, which starts with softening and ends with decay. Since the visual symptoms are more in the vicinity of the stone, the term pit burn is used. While the peel is good and does not exhibit any symptoms, the internal mass is darkened over a period in response to increased metabolic activity.

(e) Chilling injury:

An aftereffect of chilling injury is associated with the softening and browning of pulp tissues caused by enzymatic browning. This leads to the development of internal flesh browning and mealiness. The internal flesh browning is influenced by cultivar genetics and storage duration. It is reported that fruits stored at 2.2 and 7.6 °C are prone to browning initiation as compared with near-freezing temperatures [71].

(f) Mealiness:

Mealiness is associated with shorter exposure to warm temperatures post-cold storage in stone fruits, leading to a reduction in pectin methylesterase activity. As a result, the pectin becomes swollen and increases in its molecular weight. Any impact on pectin causes an imbalance in the cellular cohesion property and lessens cell integrity. This is supported by extensive senescence and tissue breakdown.

(g) Flesh Redness:

This physiological disorder manifests itself as radially appearing pink–red patches from the center in apricots and peaches [54]. It is also referred to as flesh bleeding symptoms and is an aftereffect of cold-stored stone fruits. The major reason is increased senescence or abnormal bleeding, leading to anthocyanin leaching towards the skin. A very minimal proportion of stored fruits are prone to this defect [72].

## 4. Packaging Interventions to Alleviate Storage Disorders

Temperate fruits harvested at optimal maturity stages need a protected environment to stabilize the various metabolic events and to reduce further storage disorders. This attempt was made to compile the up-to-date studies on the shelf-life extension of temperate fruits using diversified and innovative packaging technologies.

### 4.1. Conventional Packaging System

The evolution of packaging design and development has taken place over the ages and historically started with gunny bags, bamboo baskets, followed by wooden baskets, the modification of which has led to corrugated fiber board (CFB), crates, and plastic films. Often cushioning materials such as foam net, plastic liners, molded pulp trays, bubble wraps, and plastic liners are used to reduce the damage and defects. Prior to this, these were used for bulk transport but with increased consumer demand for ready retail and customized packs, there were innovations that led to various polymeric film blends and packages. This eventually witnessed a shift seen in the raw material usage and methods of film forming. The selection of these materials is influenced by the nature of the commodity, stacking strength, duration of storage, conditions of storage, cost impact, and mechanical strength of the material.

Jute-derived gunny bags, which were earlier used for fruit packing, suffered from huge product losses, as the bottom fruits were critically damaged. They were further limited by reduced stacking intensity. Bamboo baskets used for apple and pear bulk packing had the issue of breakage in long-distance transport and performed poorly with mechanical vibrations during transit [73]. Wooden boxes for packaging apples suffered with physical damage, especially on the upper and lower stacked fruits due to the sharpened edges.

Corrugated fiber board (CFB) made of paper and paper-based materials emerged as a promising alternative for long-distance transport. Being lighter in weight, coupled with ease of handling during supply chain, these added further value to the segment. They are generally used for pome fruits, apricots, plums, peaches, and cherries. Use of foams, a separator or bubble wraps, can minimize the mechanical damage. However, its susceptibility to moisture makes it a major limitation, especially in cold storage.

The transition to petrochemical-derived polymer-based packing has yielded plastic crates with better durability and ventilation benefits. Commonly derived from polyolefin (PE), polypropylene (PP), and polyvinyl chloride (PVC) with the benefit of reusability, these make it a popular choice for bulk and long-distance transport. In addition, the origin of various formats such as trays, stand-up pouches, and cups was derived suiting the commodity value and customization requirement.

Synthetic polymers from petrochemical sources form a widely used packaging matrix in the food industry. With respect to temperate fruits’ preservation, several polymers such as low-density polyethylene (LDPE), high-density polyethylene (HDPE), polypropylene (PP), and laminate blends of various polymers are investigated. Key results include maintaining the homeostatic behavior of fruits and minimizing the onset and progression of disorders. Nath et al. [74] compared polymer matrix suitability for MAP storage of pear. The results highlighted the role of PP films in minimizing physiological loss in weight (PLW). Reduced values for PLW are extremely beneficial for delayed ripening and softening progression. In addition, due to the restricted permeability of O_2_, there was an associated decay observed in PP at 16.5%. An appreciable TSS ratio was also maintained without much variation. TSS variability is one of the few quantitative indicators of several physiological disorders. The importance of perforation in packaging films was described by Mosie et al. [75], where the authors observed a reduction in PLW in non-perforated bags, which may be due to reduced oxygen availability. Contrary to this, the studies of Akbudak and Aris [76] showed a relatively lower shelf life of nectarines in PP as compared with LDPE. Every fruit must attain an equilibrium state with the receipt of gases for the trigger of enzyme action. This could be the reason for the variation in the films’ performance. A slow yet sustained reduction in polyphenols was observed in LDPE-packed apricots by Ali et al. [77], which could be due to the extended branching structure of LDPE capable of lowering the phenolic conversion-induced browning. Besides these factors such as polymer type, grams per square meter (GSM), orientation of films, methods of film forming, material properties of films, and the storage conditions play a critical role in evaluation of packaging material for fruits. Films derived from PP are an excellent barrier to water vapor and deliver an extended chemical resistance that performs equivalent to the HDPE films with the advantage of being transparent.

### 4.2. Biodegradable Polymers

The requirement of sustainability and the urge for reducing the global greenhouse gas emissions have led to a partial reliance on the potentiality of biodegradable films for packaging application. Accordingly, various sources of biopolymers have been extracted, evaluated for their material properties. The nature of biodegradable polymers are categorized as follows:

(a) Biomolecule derived
(b) Synthetically derived biopolymers from natural biomass or synthetic materials.
(c) Microorganism sourced.

To enhance the performance of biopolymers, they often require the addition of various fillers, additives, and adjuvants due to the non-performance of films on their own. Alternatively, the neat biopolymer requires certain pretreatment or modification to enhance and meet the material requirements of packaging industry [78,79,80]. Much of the biopolymer packaging finds extensive usage in MAP, active packaging technologies. They are used either as a coating application or for film development. Wax coating, widely used for the cold storage of apples, represents the lipid category of biopolymers derived from natural and synthetic sources. The greater hydrophobicity of waxes makes it a promising biopolymer in fresh fruits’ preservation. A polylactic acid-derived container was used in packaging of blueberries where the authors showed a temperature dependency behavior of the polymer in reaching steady state equilibrium. This was responsible for hastening the acid conversion in the berries. In addition, the used biopolymer was able to retard the weight loss in comparison with the commercial clam shell [81]. PLA exhibits an equivalent material characteristic of PET and oriented polystyrene (PS) and shows a melting temperature around 150–180 °C. The report provides a key insight into utilizing PLA as a reinforcement material for the biopolymers.

A promising benefit of an increased gas barrier was observed on blending whey protein with the starch films. The 80:20 blend of starch: whey protein had maintained stable respiratory rates in wrapped plums. Increases in protein tend to improve the glossiness and can provide entrapment sites for the incorporation of bioactive extracts. But a smaller increase in CO_2_ was noticed in high-temperature-stored plums (22 °C). It should also be a concern that more CO_2_ at higher temperatures can boost ethylene levels. Also, the weight loss was very minimal due to the combination effect on reducing the permeability of moisture. The storage at 3.5 °C was preventive enough to have reduction in weight loss as compared to 22 °C [82]. Biodegradable options are a great substitute in the present scenario; however, the proper selection of biopolymers with due consideration to barrier properties and appropriate blending strategy can overcome the lacuna and provide promising results. The presently used biopolymers tend to exhibit minor pores and cracks; thereby, compromising on mechanical and barrier properties. There may be chances of aroma migration between the stored environment and the food matrix. Migration studies of biopolymer with simulated food systems provides insights into the nature of polymer breakdown and the hydrolyzed products. Mutsuga et al. [83] observed the release of oligomers and dimers of polylactic acid at levels of 0.008–0.040 mg/dm^2^ which had shown a temperature-dependent rise. Similarly, first order kinetic reaction was observed for the triacetinin plasticized starch acetate films. The migration initially followed Ficks diffusion mechanism which later transformed into a non-Fickian model due to starch molecular expansion [84]. In addition, to combat these defects there needs to be a change in the fabricating film apart from solution casting methods, to increase the strength and material properties of biodegradable films. Studies have also shown the use of active ingredients and phytochemical constituents entrapped in biopolymer matrices in providing antioxidant and antimicrobial effects to the developed active packaging films [85].

### 4.3. Vacuum Packaging

Designing a protective package around fruits with the complete evacuation of air and creating a vacuum environment restricts all kind of metabolic activity in the respiring fruits. Though very popular in meat-based food segments, very limited studies have been conducted in individual packing of fresh or fresh-cut fruits. Packaging materials and storage of post-treated fruits ensures the shelf-life extension of fruits. It is of critical importance to select high barrier packaging material to restrict completely the air movement. Denoya et al. [86] studied the feasibility of using high-pressure processing with vacuum packaging on fresh-cut peaches. The study revealed that vacuum-packaged peaches almost inhibited the enzymatic browning. Since the packaging completely removes oxygen, there is the advantage of reducing the browning disorders. The vacuum packaged fruits are vulnerable to air leakage during the supply chain; hence, it becomes essential to ascertain the leakage intensity of packed commodities. Micro- and nanosized defects are not easily detected using destructive methods. Koruk and Sanliturk [87] studied an acoustic-based method to evaluate the defects of >150 μm. The air movement in the headspace of packed food created a pressure difference that was detected using a low noise microphone and converted to frequency mode. Based on the frequency limits, the faultiness in the package was detected. In addition to this, the possible risk of generating fermentation end products such as ethanol acetaldehyde leading to off flavors should not be undermined in the fruit packaging design.

### 4.4. Modified Atmosphere Packaging (MAP)

MAP is extensively used to regulate the respiratory behavior of harvested fruits. This technique makes use of gas (CO_2_ and O_2_) in synergy with packaging material permeation properties to arrest or slow down the respiration, gaseous exchange, and metabolic activity of fruits, thus increasing the shelf life. The fruit physiology, initial gas concentration, thickness of film, barrier properties of films, and storage condition play a critical role in deriving maximum benefit of MAP. Generally, a polymer capable of maintaining reduced O_2_ and increased CO_2_ is desirable to halt the respiration process and prevent microbial damage, respectively. Many of the MAPs employ the use of conventional polymers such as polyethylene terephthalate (PET), polyethylene (PE), polypropylene (PP), and polyvinyl chloride (PVC) [88]. In addition, various laminates and extruded polymers are being used with key consideration of material properties (GSM, thickness, orientation) and tensile properties.

MAP technology with PET containers was used in fresh-cut pears in combination with NatureSeal^®^. NatureSeal is a registered formulation with calcium ascorbate used in fruit preservation as an anti-browning agent. The authors observed a gradual reduction in O2 and increase in CO_2_ due to ongoing metabolism in fruit tissue. The TSSs, TA, and pH of the MAP pear were significantly not different from the control samples. The 21 day MAP stored pear slices were equivalently comparable with 12-h prior sliced pears for aroma, texture, and flavor; however, the color change was slightly observed [89], which could be due to internal gas concentration leading to browning. There was a significant color change effect (L*, a*,b*) in MAP fruits after 14 days of storage which implies that the protective barrier was able to maintain the appreciable permeation and had not allowed for CO_2_ buildup up to 14 days. Cheng et al. [90] provided concrete evidence on effect of MAP LDPE film thickness (10 μm, 30 μm). Film with higher thickness permitted the stocking of more CO_2_ (1.6%), thus accelerating the core browning disorder. At the other end, the 10 μm film was excellent enough in reducing the browning and phenolic release. Generally, the CO_2_ inhibition tries to follow any one model—no inhibition, competitive inhibition, non-competitive inhibition, and uncompetitive. In a no inhibition type and in contrast to the above results, Oguz-Korkut et al. [91] showed the lowest color change under a CO_2_ concentration of 5% which signifies the non-dependency on CO_2_. To avoid the possible hypoxic conditions, researchers have resorted to the use of semi-perforated packaging materials. Caleb et al., 2018 studied mathematical modeling for micro-perforated films of fruits and vegetables used in packaging [92].

The PP + LDPE laminate showed a relatively lower time for equilibrium attainment of O_2,_ which confirms the better retention of respiratory behavior in pear in the selected laminate structure [93]. Immediately after packing the desired fruits in the packaging material with a defined gas concentration, the headspace availability above the fruits tries to establish a mass balance. This could be the diffusion of respiratory byproducts from core tissue to periphery and to the external surroundings via the film. Several researchers have predicted the response behavior of MAP-packed fruits by physicochemical analysis that can well predict their shelf life [94,95].

**Figure 3:**
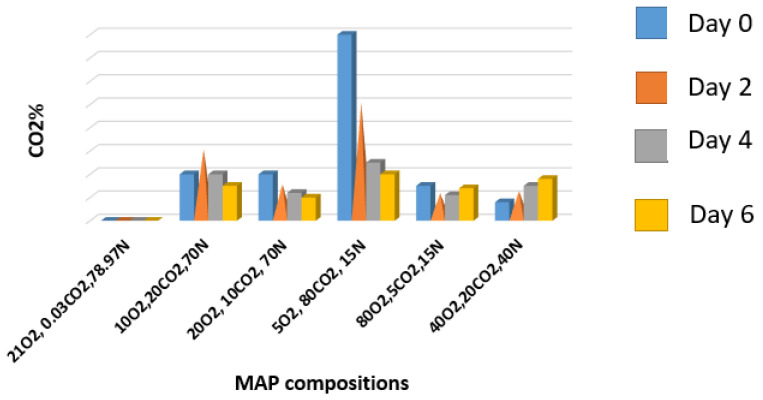
CO_2_ reduction trend in the selected LDPE polymers.

The passive MAP often takes a certain time to establish equilibrium and reduce the O_2_ gas content, while the active MAP continuously tries to monitor the desired gas concentration either by removing or injecting the desired concentration of gases. Muftuoğlu et al. [96] studied the hurdle approach by preliminary treatment with commercially available antioxidants and following active and passive packaging. Both filled PP trays sealed with biaxially oriented polypropylene (BOPP) were stored in 4 °C with 75% RH. The use of BOPP has given the advantage of better retention of gas in the fruit side. It was observed that the MAP technique was able to bring an appreciable lesser reduction in chroma index in both coated and non-coated apricots as compared against the synthetic pretreatment of antioxidant. There was hardly a mass loss of 0.2–1% as compared to the control 57%. Careful selection of films and combinations can potentially act to reduce the cost impact of various additives. LDPE-based MAP in apricot showed comparatively higher permeation for CO_2_ than the O_2_ (Figure 3). In the selected treatments, as high as 80% CO_2_ was drastically reduced to <25% which was responsible for causing a lower vapor pressure deficit and thereby preventing the weight loss. The residual CO_2_ was responsible for precipitating the cellular pectin and preventing the hardness adversity.

Recent innovative approaches in MAP include the combination approach using physical and chemical techniques [97]. Serrano et al. [98] explored the utilization of essential oils in cherry MAP preservation. The essential oil pretreated cherries had retained green stem and reduced microbial proliferation. This shows the antioxidant and antifungal benefits of utilizing essential oils. Similar studies on the use of ozone [99] bioactive extracts [100] and preheat treatments were observed [101].

Dynamic gaseous adjustment in honey peach was studied using laser microporous polyethylene films (LMPF) [102]. The pre-fumigated peaches with 1-methylcyclopropene (1-MCP) were packed in LMPF. This combination treatment was able to retain the concentration of ascorbic acid and maintain titratable acidity. The use of 1-MCP has a proven record in reducing the antioxidant enzymes and inhibiting or delaying ethylene regulation and thereby delaying the softening or senescent-induced effects. Here, in this study, the combination with LMPF was able to show a reduced firmness loss in addition to being able to reduce the concentration of various antioxidant enzymes. Higher loss of firmness is associated with the pectin degradation in exposure to CO_2_; in this study, the laser-perforated films were capable enough to allow for a rapid CO_2_ release. Generally, the laser perforation technique is used in both synthetic and biodegradable polymers to increase the gas permeability [103]. MAP utilizes oxygen in the range of 1–5% depending on the respiratory potential of fruits being packed; however, this higher oxygen ratio can have the possibility of inducing fermentation, reducing the aroma synthesis, and off-flavor development. Hence, research on super-atmosphere packaging with higher O_2_ is being carried out to derive the positive benefits. Allende et al. [104] investigated the use of super-atmosphere packaging (80 kPa O_2_) and CO_2_-enriched packaging (10 kPa CO_2_) on strawberries MAP. Though both the processes maintained the required firmness over storage period, there was an observed biochemical change in each technique. While super-atmosphere O_2_ had promoted the oxidation issues leading to degradation of vitamin C and phenolics, the CO2-enriched MAP was successful in preventing the psychrophilic growth but negatively influenced the flavor development by forming various fermentation intermediaries. Another issue of MAP is the CO2-induced growth of anaerobic microbes in the food matrix. Intervening with precise gas modification, enabling the creation of equilibrium moisture content, or developing microporous MAP can lower the microbial incidence [105].

### 4.5. Edible Coating

**Figure 4:**
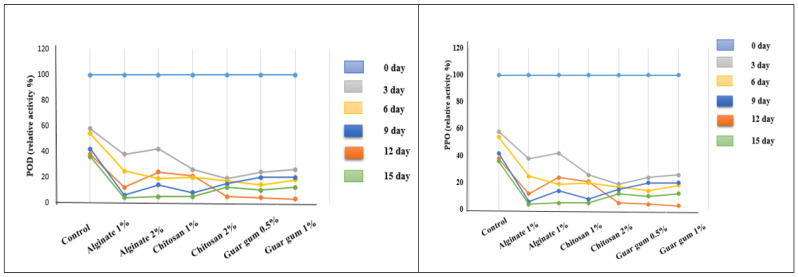
Effect of selected coating treatments on the enzyme activity of apricots.

Edible coating technology is widely recognized for enhancing the appearance, quality acceptance, and marketability of the fruits. Generally, biopolymers such as polysaccharides, proteins, and fats act as a semipermeable matrix in modulating the gaseous exchange. Proteins and polysaccharides, owing to their brittleness, need plasticizers to enhance the flexibility of polymers. But an excessive dosage of plasticizer can irreversibly increase the vapor transmittance properties and can affect the shelf life. Morsy and Rayan [106] explored the feasibility of using polysaccharides such as gellan gum (G), alginate (A), and chitosan (C) in edible coating formulation. The polymers were able to increase pH by 3.45, 4.17, and 2.74% for A, C, and G films upon storage for 15 days. The A films at 2% were able to prevent the TSS increase and showed 12.8 °Brix as compared with control (14.4 °Brix) with a corresponding lowest reduction in titratable acidity. This also shows the helical confirmation of alginate in becoming a better barrier for the gas movement and thereby reducing the respiration and other metabolic events. Alternatively, the use of C was successful in retaining the ascorbic acid by preventing the oxidation effect. The 15th day stored apricot showed C (13.68 mg/100 g) ascorbic acid as against the A (12.48 mg/100 g) and G (11.56 mg/100 g). Irrespective of the polymer type, all showed a significant reduction in PPO and POD enzyme activities (Figure 4).

**Figure 5:**
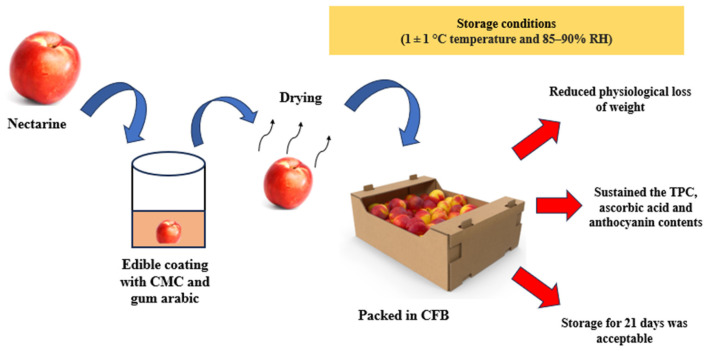
Effect of edible coating and storage in CFB on Nectarine cv. Snow Queen.

Stone fruit are known for their shorter shelf life. An aloe vera-based coating in combination with rosehip oil was able to reduce the respiratory rate and ethylene release in plums and prunes. Nectarine shelf life was extended for 28 days in a sequential strategy of hydrocolloid coating followed by corrugated fiber board (CFB) stored at temperatures (1 ± 1 °C) and RH of (85–90%). The synergy combination prevented very well the activity of pectin methylesterase (PME) [107] (Figure 5). Increase in respiratory output from the resting fruits triggers the release of several cell wall degrading enzymes that try to solubilize the pectin middle lamella allowing for cellular leakage causing shriveling ultimately leading to softening. Coating strategies act as a preservative principle and adhere to reduce or lower the enzyme release. Measurement of electrical conductivity as an indicator of membrane leakage was studied in sweet cherries. An edible coating with shellac wax was able to reduce the conductivity values and thereby retard the respiratory changes [108]. A 0.5% chitosan coating on sweet cherry was linked to maintaining stability in chroma values and delayed softening. The excess concentration of 0.75% had a negative effect in reducing chroma, mainly due to high-concentration-induced brittleness formation [109].

Various additives such as antioxidants, antimicrobials, anti-browning, and firming agents are incorporated during edible coating to increase the effect on shelf life. The compatibility of incorporated additives determines the success of intended delivery. Incorporated anti-browning agents were not that effective when used with hydrophilic biopolymer (alginate and gellan gum) coatings in increasing the antioxidant effect and water vapor barrier properties in fresh-cut apples [110]. At the other end, addition of lipophilic sunflower oil drastically increased the barrier properties of the coating. Not all the coating solutions will be a perfect fit for the fruit matrix. The additives and polymers need to be optimized for the concentration and responses should be evaluated with response to shelf life and consumer purchase decision. The advantages of cross-linked effects of apple peel polyphenol and chitosan contributed to the reduction in weight loss of coated strawberries [111]. The stereochemistry and reactivity kinetics of selected biopolymer and additive need to be critically appraised prior to coating operations. Lipid-based coatings include the use of waxes that are hydrophobic and are a renowned material for water barrier effects. An increased usage of diversified waxes is being deployed for preservation of fruits. Important wax materials include bees wax, candelilla wax, and carnauba wax that differ with the raw material, wax extraction method, wax composition, method of wax coating, and storage of wax-coated fruits. In many of the coating experiments, it is common to observe the calcium chloride dip to increase the firmness and reduce the extent of softening [112]. CaCl_2_ assists in strengthening the cell wall fragments and thereby the degradation and membrane collapse is delayed. The importance of storage temperature in coated fruits was shown in the studies of Thakur et al. [113] where rice starch–fatty acid ester-coated apples stored at 5 °C were able to retain the TSS gain and evidenced the reduction in diphenyl picrylhydrazine (DPPH) scavenging assay. A reduced value for DPPH scavenging implies minimal increment in the respiratory release profile of fruits. In combining the coating followed by packaging approach, the apple wedges with anti-browning agents (4-hexyl resorcinol and ascorbic acid) and edible coatings (Aloe vera gel and carboxymethyl cellulose (CMC)) packed in a PP box showed a better whiteness index with reduction in browning reactions. This is confirmed with lower values for POD and PPO enzymes. It is of paramount importance to study the temperature profile of coatings on fruits as the fruits often tend to change in extremes of temperatures with respect to their metabolic and respiratory events.

Whilst the edible coatings on temperate fruits are a very successful procedure in extending the shelf life, the issues relating to certain innate microbes and pests may not be overcome. As the fruits are surface coated, there can be no guarantee of pest or microbial infection in the inside parts of the fruits. Adapting a non-destructive method for quality inspection can add value to the coating studies. Moreover, since these are edible in nature and go for direct consumption, regulation on migration and its edibility needs to be properly checked prior to entering the consumer market. The migration factor of coating materials essentially is influenced by the thickness of coating, the material selected for coating, its molecular weight, and cross-linking impacts [114]. Various additives such as nanomaterials, essential oils, and plant extracts often differ with their levels of allergenicity or toxicity to human subjects; therefore, there should be critical evaluation of safety and efficacy of dosage of these fillers before synergizing with the coating matrix [115].

### 4.6. Metal Oxide-Coated Multilayered Packaging System

To enhance the barrier characteristic of polymeric films, use of metal oxides is increasingly investigated and has resulted in the use of silicon dioxide (SiO_2_), aluminum dioxide (Al_2_O_3_), titanium dioxide (TiO_2_), and zinc oxide (ZnO). Earlier, the use of aluminum foil was regarded as the best barrier polymer but unfortunately it could not be used in microwave-assisted thermal sterilization due to its inherent nature of reflecting the microwaves [116]. Hence, various metal oxides emerged as alternatives in this segment for fulfilling the packaging of thermally processed foods and exhibit desired transparency. The combination approach of selected multilayers can result in a synergistic impact as against the individual poor performance. This area is rapidly increasing its use in the functionalization of blended biopolymers. The metal oxides can sometimes act as a antimicrobial agent in the blended polymer system [117]. Generally, these oxides can be surface coated via physical vapor deposition, chemical vapor deposition, reactive thermal evaporation, grafting, pulsed laser deposition, atomic layer deposition, spray coating, and the plasma polymerization principle. Struller et al. [116] studied the aluminum oxide-coated synthetic polymer films (BOPP and PET) and observed the critical role of base polymers in producing a high barrier effect. The cracks and pores in the neat BOPP had led to a decrease in barrier properties and impaired the nucleatic growth of aluminum molecules. BOPP is a non-polar polymer which differs in the adhesion and blending properties; this study also highlighted the role of high surface energy polymer which increased the barrier properties as compared with a sole polymer. In conjugation to the pre- and post-plasma treatment, OTR of 0.83 cm^3^ (STP)/(m^2^ d bar) was observed which was equivalent to the standard PET polymer property. Understanding the due importance of surface chemistry and adhesion properties can increase the chance of fabricating a barrier-improved film.

The success of multilayered films lies in the polymer chemistry, method of coating metal oxides, and the post-processing technique employed. Parhi et al. [118] provided conclusive evidence on the performance of metal oxide (SiO_2_, Al_2_O_3_)-coated PET during the retort processing (121 °C) for 20 and 30 min. The authors observed the change in enthalpy of metal oxide-coated polymers due to formation of cracks and pinholes which eventually caused shrinkage of polymer and increased the permeation rates for O_2_ and water vapor. An overlayer of polyacrylic acid was successful in preventing the shrinkage.

Devising barrier packaging that can sustain the novel pressure-assisted sterilization sometimes becomes challenging due to inability of available polymers to sustain a higher pressure potential. To augment this area, Al-Ghamdi et al. [119] highlighted the role of aluminum oxide PET (AlO_x_-coated PET//AlO_x_-coated PET//PA//CPP) for pressure sterilization of avocado puree. The metal oxide-coated polymer exhibited a greater barrier for OTR and WVTR with an enhanced performance for former movement. The pressure induced also had an obvious impact on molecular restacking and increasing the crystallinity and thereby created a torturous pathway for gas movement. In support, Patel et al. [120] observed a greater water barrier potential in three-layered metal oxide-coated films which could relate to the molecular pattern and hydrophilic/hydrophobic behavior of the polymers. The lowered WVTR was preventive enough to reduce the weight loss of purple mashed potato during storage. But the polyacrylic acid-coated polymer and aluminum foil, by virtue of its greater O_2_ barrier properties, had a comparatively lower loss of sensitive vitamin C, which underscores their light and O_2_ barrier properties. To further strengthen the protective role of metal oxide-coated polymeric performance in food systems, Albahr et al. [121] evaluated the AlO_x_-coated multilayered PET films in MATS for beetroot, cabbage, and carrot models. The basis of selection of food matrices is its relatively high concentration of betalains, anthocyanins, and carotenoids with differing solubility and polarity. The polymer was successful in maintaining the color stability during storage in addition to minimizing the weight loss occurring due to molecular movement.

Further, these metal oxides can be nano-reduced (MONPs) and utilized in the bio-nano-composite films which further enhances the barrier properties. Nandhini et al. [122] exclusively provided a review of the various nanometal oxides that can be used in the food systems. TiO_2_ with Zn dopant exhibited antimicrobial activity against *Staphylococcus aureus* and *Escherichia coli* and extended the shelf life of plums by 16 days [123]. Further, ZN-based MONPs based on their piezoelectric nature can be utilized in the sensor development [124]. 

### 4.7. Active Packaging

Active packaging represents a subset of innovative packaging technologies where the vicinity environment of the commodity is continuously monitored and modulated using emitters, absorbers, and scavengers. There is a deliberate use of technological constituents in the headspace of the commodity (sachets) or the packaging matrix (lid attachments, label placing). Active packaging is used extensively in all domains of food products, encompassing fresh fruits, vegetables, meat, bakery, and other convenience-packed products. Temperate fruits being climacteric in nature are continuously under metabolic stress that leads to emission or reception of various gas constituents, which further has continued effects on metabolic progression affecting the ripening chain and storage stability [125].

**Figure 6:**
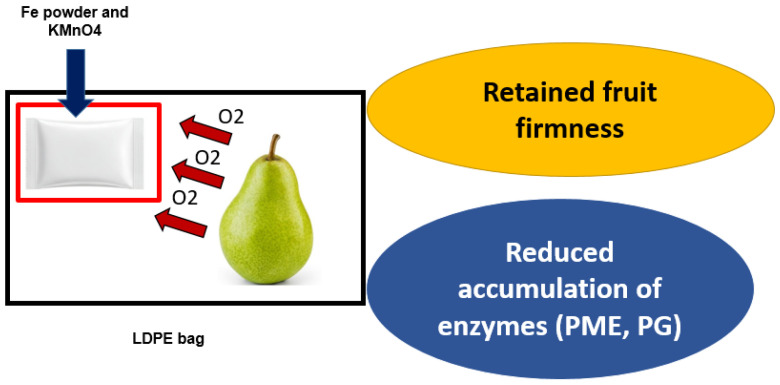
Active packaging of pear fruit (cv. Bartlett).

Active packaging using oxygen scavenger (iron powder (Fe)) and ethylene scavenger (potassium permanganate (KMnO4)) in sachet forms were placed in LDPE-packed pears and was evaluated for physicochemical changes (Figure 6). The active ingredients were able to extend the shelf life of pear fruits by maintaining the firmness and reduced the PLW. Even after exposing the cold-stored pear to ambient conditions (9 days), the fruit maintained well with a mere 7.9% weight loss. A 15% Fe-powder sachet in combination with high thickness LDPE (50.8 μm) was able to minimize the oxidation of ascorbic acid and reduce the climacteric maxima attainment. After 90 days of cold storage, the active-packed pear reached 29.3 CO_2_ ng/kg/s indicating a slower metabolic activity. The combination of Fe powder with KMnO4 was successful in removing the free oxygen to form Fe_2_O_3_ and oxidize released ethylene to CO_2_ and H_2_O. The Fe powder demonstrated the activity of lowering or arresting photo oxidation, enzymatic oxidation, and fatty acid oxidation by removing the free oxygen. In comparison with free Fe powder, micro- and nano-reduced Fe powder had an excellent scavenging effect [126]. While designing concentration of O_2_ scavenger, it is essential to understand the respiratory behavior of fruits and permeation behavior of packaging materials coupled with absorption rate and absorption capacity of scavenging material. Overuse of Fe powder despite a lower O_2_ content in food matrix can lead to accidental consumption causing health hazards. On the other hand, the maximal adsorption capacity of the active ingredient and sachets needs to be optimized to increase the process efficiency. Gaona-Forero et al. [127] deduced the equilibrium adsorption capacity of the moisture adsorber by using Equations (1) and (2).

(1) $$C=\frac{W(eq)-W(in)}{W(in)}$$

where C is the equilibrium adsorption capacity of the adsorber (kg/kg); W(eq) is the equilibrium adsorber weight; and W_in_ is the adsorber weight at initial time. Further, the adsorption rates were calculated using Equation (2).

(2) $$\;\;\;\;\;r=\frac{W\left(ad\right)t+1-W\left(ad\right)t-1}{W(delta\;t)}\;\;\;\;\;\;\;$$

where r is the adsorption rate at time t (kg/kg/day); W (ad)t + 1 is the adsorber weight at a time after ‘t’; and W(ad)t-1 is the adsorber weight at the time before ‘t’.

Other commonly used O_2_ scavengers include ascorbic acid [128], tocopherols [129], glucose oxidase [130], phenolic acids [131], platinum based [132], and unsaturated hydrocarbon based [129]. Various plant-derived metabolites such as phenolic acids, flavonoids, essential oils, and plant bioactive components, are investigated as active agents in the fruit preservation applications. To circumvent the storage pathogenic infestation, da Rocha Neto et al. [133] developed β cyclodextrin inclusion complex (IC) of essential oil that was placed suitably in a perforated double bottom system. However, penicillium-inoculated apples stored in double-perforated IC packages did not eliminate the fungal growth, but had significantly reduced lesion development with respect to the control. The essential oils vapochromic initiation is triggered upon achievement of threshold RH condition, and due to the delay in attaining the required RH condition, there was some microbial growth observed. The added essential oil of star anise had the positive effect of inhibiting the ethylene release. However, the performance of this active packaging in real time conditions needs to be explored with key considerations to temperature and time of storage. Lopez-Gomerez et al. [134] showed that the carvacrol release from the embedded film showed first order and n-order kinetics (Equation. (3)). The rate constant varied from 0.0047 to 0.0155 with R^2^ > 0.7. Further, the release profile showed a RH-dependent behavior. The 60%RH showed release of 0.70  ×  10^−2^ and 0.48  ×  10^−2^ 1/day and increased to 0.926  ×  10^−2^ and 0.833  ×  10^−2^ 1/day for the 95% RH stored sample.

(3) lnC=lnC0−k

where ln C is the carvacrol concentration; t is the storage time; C_0_ is the initial carvacrol concentration; and k indicates the rate constant.

An interesting insight from the report of Navarro-Martínez et al. [135] showed that the vapor citral concentration above 16.7 μL/L did not have any significant impact on ethanol reduction which would be due to binding of enzymes involved in its synthesis. The compatibility of essential oils with encapsulating matrix and prerequisite for release needs to be critically understood to increase the scalability of this concept. Synergy studies on equilibrium MAP with antimicrobial pad system was successful in retaining the vitamin C and titratable acidity in cherries and strawberries. The retention of acidic constituents also had the concurrent effect in reduced loss of anthocyanins [136].

Placement strategy of cinnamon essential oil-loaded active packaging in macro-perforated PET trays and self-adhesive labels was designed by Montero-Prado et al. [137] to study the storage behavior of peaches cv. Calanda. It was observed that a label attachment at the top space of fruits was comparatively better in reducing the PLW and maintaining firmness. In addition, the lipoxygenase (LOX) enzyme concentrations were significantly reduced in labeled packaging material, indicating the lower enzymatic activity in the storage peaches.

Recent trends in the shift towards the bio-based packaging arena have compelled the research community to rely on natural polymers for the active packaging applications. A biodegradable paper-based active packaging blend (potassium sorbate, potassium meta bi-sulfite (KMS), and sodium permanganate) was useful in preventing the incidence of *Candida pelliculosa* on plums and peach, while minimal TSS variation was observed, which was near the fresh fruit. On the contrary, the optimization studies were required to blend the active materials without impacting the mechanical properties of coated films [138].

### 4.8. Intelligent Packaging

Here, the precise monitoring of quality status is achieved with the use of sensors, indicators that can receive, convert, interpret, and provide real time information to the consumers [139]. Since fruits typically move through production to distribution channels via refrigerated or cooler mechanisms, any disruption in the supply chain can initiate spoilage and leads to resource wastage. Freshness indicators, a category under intelligent packaging, is of particular interest to fruits and vegetables that can sense the ethylene trigger and be communicated to the end user. The response can be visual color change indicator or sensing of specific marker analyte either on the package or in the microenvironment of the fruit. While indicators generally provide qualitative or semi-quantitative information, the sensors are known for accurate quantification in the observed analyte. The generated data can correlate with spoilage, senescence, and metabolite leakage induction. The inputs could be changes in firmness, ripening, and chemical changes (aldehyde, volatiles, ethanol) and output could be monitored through optical, electrochemical, or chemical modes.

The Ripesense^®^ sensor, for instance, is used for ripening status colorimetric detection by sensing aromatic volatiles. The color changes from green to yellow signify the onset and progression of ripening leading to senescence. This is currently being used as a non-destructive method in ripening prediction in pears and research studies are being conducted to expand the potential use in other fruit crops.

Radiofrequency identification tag (RFID)-based quality monitoring was used by Vergara et al. [140] where a metal oxide (tin and tungsten)-based semiconductor was used as a reader tag for fruit quality monitoring. RFID tags are known to record data as large as 1 megabyte [106]. The precise monitoring of gases (ethanol, acetaldehyde, and ethylene) was conducted with prior database generation. The fabricated tag was successful in detecting the alarm level for the designated gases. Data carriers hold tremendous potential in larger scale supply chain operations that assist in better inventory management. Other commonly used data carriers include Quick Response codes (QR code), bar codes, and near field communication (NFC). A more practical approach in intelligent packaging was conducted by Liu et al. [141] where data collection was performed during cold chain logistic using multisensor approach in ‘Korla’ fragrant pear fruit where the quantification of temperature, humidity, ethylene, CO_2_, and O_2_ concentration was performed in correlation with physicochemical parameters. The obtained data sets when subjected for back propagation neural network gave a 97% classifier accuracy. In this way, the fluctuation during supply chain of fruits helps in better understanding of post-harvest physiology. In a similar line of work, Zhang et al. [142] and Qiao et al. [143] developed freshness prediction model in sweet cherries and strawberries, respectively, using backpropagation neural network.

### 4.9. Nano-Packaging

Use of nanomaterials owing to their size and immense material functions can aid in reducing the various petrochemical derivatives and additives. Nanomaterials with a size dimension of 1–100 nm possess unique electrical, thermal, magnetic, and optical properties. The field of nanomaterial chemistry and technology can potentially make a change in the future of food packaging. This is because the nano-sized materials in the form of films have the equivalent or better characteristic properties for material, thermal, and barrier, in addition to being antimicrobial and antioxidant.

Nano-packaging is an extrapolation in the use of packaging materials with the specific inclusion of nanomaterials. The sources can be either naturally derived or synthetic with chemically being organic or carbon derived or inorganic and of a varied dimension (1D, 2D, 3D) and orientation [144]. Gardesh et al. [145] performed the chitosan nanoemulsions on apple and found that they exhibited an antioxidant nature by reducing the enzyme activity, respiration, and ethylene release. The effect also showed a concentration dependency, with 0.5% provided greater results. Nano-calcium and calcium chloride applied at the pre-harvest stage were able to reduce the concentration of various cell wall degrading enzymes [146]. Nanomaterials support active packaging by inclusion as films, composites, or coating functions, while the intelligent packaging contributes its use as a sensor element in detecting key gaseous/aromatic metabolites. Bactericidal action of silver nanomaterials in packed peaches was studied by Kaur et al. [147]. In addition, there are experiments on the use of nano-zinc, nano-titanium, and nano-silica derived preservation applications [148]. A list of various advances in packaging technologies used for temperate fruit packaging is provided in Table 1.

## 5. Challenges and Potential Limitations of Advanced Packaging Interventions

Though the benefits of biodegradable packaging are encouraging, an inherent number of concerns do arise in its means of degradation and the cost involved. There is no harmony in the definition of biodegradation, which may end up in microplastic formation in longer degradation period. In addition, the life cycle analysis of some of the biodegradable polymers is alarming due to a mismatch. An increase in biodegradable polymer production should not cause the food sector any problems, as both will be competing for limited resources [172]. Lack of standardized and cost-effective manufacturing pathways for biodegradable polymers has caused the issue of pseudo-biodegradable plastics as some manufacturers blend the synthetic with the bio-based polymers [173].

MAP is evolving and represents a highly investigated packaging segment that needs further refinement on the economic feasibility. The presently used system is more specialized, requiring different concentrations of gases with trained staff handling. A potential lacuna in the supply chain is the lack of stacking, as it may endanger the gas composition. This may significantly increase the aspect of logistic costs [174]. Edible coating technology, on the other hand, is highly variable, subject to the type of fruit and the selection of coating materials. A lack of homogeneous and uniform coating solutions can make the process further cumbersome. Smart packaging systems, including active and intelligent, have revolutionized the modern packaging roles and functions but are yet to deal with the onsite problems. Active packaging with sachets requires another polymer to pack and thereby increases the plastic usage. Many of the sachet additives cannot be used in liquid foods thus posing difficulties in the versatile usage. Intelligent packaging is still in the infancy stage which requires a lot of thorough investigation on the sensitivity and the cost impact involved.

The field of nano-preservation and packaging holds promising potential; however, the inherent issue of the possible migration of nanomaterials into food and the environment cannot be neglected. The critical aspects of size, penetrating potential, and the surface-active behavior of NP are playing a crucial role in the assessment of health risk and ecological toxicity. Though some of the investigations have proved the role of nanomaterials having been migrated into food matrix, very limited studies exist for its direct role in environmental impact. Song et al. [174] observed temperature- and time-dependent migration of Ag NPs into the simulated acidic food system. Echegoyen et al. [175] further deliberated on this and evidenced the release of Ag NPs in microwavable containers. The migration of NPs and their health impact at the permissible limits is still a debatable issue. This is because, on the one hand, there are studies on the carcinogenic effect of some of the nanomaterials that showed the possible buildup of nanomaterials in human organs owing to their insolubility in the systemic circulation. On the other hand, there are certain policy and regulatory hurdles in maintaining consensus in the migration limits. The reason is the lack of human studies relating to the potential toxicity of NPs and their bioaccumulation, limited risk assessment reports, and scarcity of information on actual food system assessment [176,177].

## 6. Conclusions

Fruits, being a perishable and actively respiring commodity, need a well-designed and comprehensively thought-out packaging approach. The selection of suitable packaging has to manage a critical balance between volume, quality, and the end consumer. Though there is a transition towards the use of advanced packaging materials in relation to the biochemical composition of the fruit matrix, it requires further in-depth investigation studies with respect to its safety, toxicity, and residual effects. The concept of nano-packaging and edible coatings is still in laboratory conditions, and needs a scale up with respect to technological adaptability and scale-up factor. This advancement, particularly the use of edible nano-packaging, needs to be the subject of toxicological studies, as many of the nanomaterials pose a risk to human health. Resorting to advanced fabricating techniques can reduce the lacuna associated with many of the polymer designs. The intelligent packaging is in its infancy stages and needs through attention and focus with the application of artificial intelligence and machine learning algorithms to expand its traceability and safety during fruit supply chain. Further, the tracking of nutritional composition along the supply chain can add value to the fruit and increase its marketability. Use of smarter gadgets, although advantageous, lead to greater waste generation and necessary steps for culminating or effective disposal need to be studied. In addition, the effective utilization of agro residues to derive active ingredients capable of exerting functional preservative effects is a welcome step. This review has tried to gather most of the biochemical events occurring in the fruit post-harvest and before consumption that could be of tremendous interest while designing packaging materials. Most of the biochemical events are a cascading set of reactions catalyzed by ethylene generation and progression. The formulation of semi-permeable film encompasses the respiratory gases’ kinetics and diffusion studies in conjugation with film barrier properties. The future of packaging should focus on cross responsive sensor arrays capable of detecting various metabolites so better information regarding fruit status is gathered.

## Figures and Tables

**Table 1:** Advanced packaging technologies for quality improvement and shelf-life enhancement in selected temperate fruits.

S.NO	Packaging Material/Technology Description	Fruits	Properties of Film/Coating Material/Storage Conditions	Effect on Fruit Matrix	Reference
1	Polysaccharide based edible coating with NAC# and CC and packed in PP trays	Fresh cut apples	PP permeability in trays was ✓ O_2_—110 cm^3^/O_2_/m^2^/bar/day; ✓ CO_2_—500 cm^3^/O_2_/m^2^/bar/day	✓ Reduced ethylene production ✓ No effect on color and firmness during storage period ✓ Shelf life extended for 2 weeks at 4 °C	[149]
2	Alginate, pectin and gellan gum edible coating with ascorbic acid and CaCl2 with apple fiber and inulin	Fresh-cut Golden Delicious apple cubes		✓ Stable color and firmness at 4 °C for 16 days ✓ Acceptable organoleptic property	[150]
3	Nanoemulsions of sodium alginate with lemon essential oil	Fresh cut Fuji apples	✓ Average droplet diameter—62 nm ✓ Average droplet diameter—62 nm ✓ Viscosity—< 99 mPa. s	✓ 0.1% LEO had lower growth of psychrophilic microbial growth ✓ No impact on firmness for 14 days stored at 4 °C ✓ Higher concentration (0.5%) of LEO had browning effect	[151]
4	Edible coating with Semperfresh^TM^, Niprofresh^TM^, and lac-based	Plums cv. Santa Rosa	Storage for 35 days 2 ± 1° C and 85–90% RH	✓ Lac-based coating showed 55% retention in firmness at end of 35 days of storage at 2 ± 1° C and 85–90% RH ✓ Delay in color change with reduced hue angle values ✓ Lowered rate of ethylene release and respiration in lac-based coating ✓ Higher antioxidant values at the end of 35 days in lac-based coating 16.21 μmol Trolox/gFW	[152]
5	Composite edible coating with hydroxypropyl methylcellulose/beeswax with antifungal agents—sodium methyl paraben, sodium ethyl paraben, and potassium sorbate	Plums cv. Friar	Storage for 22 days at 1 °C + 5 days at 20 °C	✓ No appreciable changes with respect to control of weight loss parameter ✓ Firmness retention in coated samples stored for 22 days at 1 °C + 5 days at 20 °C was comparatively higher than others ✓ Increased titratable acidity in coated samples ✓ Lowered CO_2_ evolution rates in coated samples	[153]
6	Composite Edible coating of buckwheat starch/xanthan gum/lemon essential oil	Plum	2:1 ratio of BS and Xanthan gum with 1.25% LEO had the highest antioxidant (73.3%) and antimicrobial efficiency	✓ Reduced water loss, and stable pH and TSSs maintained at 20 days of refrigerated storage	[154]
7	Edible coating of sodium alginate, and methyl cellulose	Peach	Methylcellulose: Ethyl alcohol (0.03:1 mixing) Sodium alginate: water (0.02:1) Storage at 15 °C for 40% RH	✓ Reduced moisture loss in MC-coated samples ✓ Reduced respiratory rate and delay in attaining respiratory peak climacteric ✓ Better firmness retention in MC-coated fruits	[155]
8	Edible coating of Rhubarb extract/Sodium alginate	Peach cv. Baihua	1% sodium alginate with 50 mL rhubarb extract S Storage 7 days at temperature of 28 ± 1 ◦C and 90%RH	✓ Coated fruits showed a lower weight loss ✓ Maintained firmness, and increase in TSSs was seen ✓ Delayed the respiratory rate in coated fruits ✓ PPO activity and MDA concentration in coated fruits was 37.1% and 20.8% lower than control fruits, respectively	[156]
9	Edible coating (chitosan, sodium caseinate, pectin) followed by MAP (PP trays with pore diameter of 100 μm)	Fresh-cut Nectarine	O_2_ permeability of trays—680 ± 24 cm3/m2/24 h/ASTM CO_2_ permeability 2.5 ± 0.1 cm3/m2/24 h Storage for 7 days at 3 °C	✓ No significant impact on respiratory rate reduction and weight loss ✓ Increased firmness in range of 2.96–3.11 N/mm in coated and stored peaches ✓ No appreciable increase in shelf life was observed	[157]
10	Edible composite coating of chitosan, tannic acid, and bees wax	Peaches	0.5%, 0.5% and 2% incorporation of chitosan, tannic acid and bees wax Variation in stability of coating emulsion was observed DPPH scavenging activity of 87.64% Storage at 25 °C for 9–10 days	✓ Reduced weight loss and non-significant firmness maintenance (4.453 N) ✓ Decrease in pH and titratable acidity	[158]
11	Edible composite coating of soy protein isolate SPI/HPMC/olive oil/potassium sorbate PS	Pear cv. Babughosha	Coated fruits stored in plastic boxes at temperature (28 ± 5 °C) and RH of (60 ± 10%)	✓ Increase in olive oil negatively influenced weight loss ✓ Reduction in pH was observed ✓ Optimized ratio of 5%, 0.40%, 1% and 0.22% was obtained for SPI, HPMC, olive oil, PS	[159]
12	Edible nanoemulsions composite coating of alginate/TiO_2_ nanoparticles/mousami peel extract	Peach	_	✓ Significant reduction in PPO, titratable acidity was observed ✓ Lowered yeast and mold count ✓ Retained color index in coated fruit	[160]
13	Edible composite coating using free radical grafting with caffeic acid, chlorogenic acid with chitosan	Saimati apricots	Grafting was confirmed by peak absorbance at 210–500 nm Grafting reduced the crystallinity of chitosan Coated fruits stored for 35 days at 1 °C in 80–90%RH	✓ Chlorogenic acid-grafted chitosan was able to maintain the firmness, TSSs, and TA. ✓ Delayed rate of respiration was seen at 0.5% chlorogenic acid grafted chitosan	[161]
14	Edible coating comparison of chitosan and chitosan nanoparticles	Apricots	✓ Chitosan NP had 16.4 nm diameter ✓ Chitosan NP had inhibitory zone of 20.0 to 25.5 mm and 17.3 to 19.0 for bacterial and fungal assay ✓ Storage 30 days at 5 ± 1 °C and 9 days at 25 ± 3 °C	✓ Lowest weight loss of 6.75% and 8.48% in room temperature-stored and cold-stored 1.5% chitosan NP-coated fruits ✓ Lowered % of visual decay ✓ MDA of 1.43 mol/g and 0.49 mol/g in 25 and 5 °C stored coated apricots	[162]
15	Electro-spun nanofiber of cinnamon essential oil with polyvinyl alcohol and 1-methylcyclopropene	Apricots	_	✓ Decreased accumulation of H_2_O_2_, MDA, SOD, CAT, POD ✓ Lowered weight loss rate and TSSs ✓ Reduced the firmness deterioration	[163]
16	Bio-nano-composite based MAP	Strawberry	✓ PLA/montmorillonite Cloisite 20A/Triacetin Storage at 4 °C for 23 days	✓ Reduced quality loss ✓ Lower weight loss and firmness retention in 10% O_2_ + 15% CO_2_ + 75% N_2_ gas composition MAP	[164]
17	Electrospray coating of cellulose-based active packaging with fulvic acid and sericin	Pear	Storage at 7 °C for 90 days	✓ Coated fruits with fulvic acid retained color and texture with lower microbial load	[165]
18	Active packaging with montmorillonite and cinnamon leaf essential oil in chitosan bag	Pear	_	✓ 0.75% and 10% incorporation of cinnamon leaf essential oil and montmorillonite had reduced weight loss and improved color	[166]
19	MAP with microperforated PP bags (0.55 mol cm/cm^2^ atm day and 0.30 mol cm/cm^2^ atm day)	Navalinda sweet cherries	Storage for 8 days at 4 °C continued with 4 days at 8 °C	✓ Reduced the deterioration decay, retained firmness and reduced intensity of softening, slower darkening of skin color in 0.30 mol cm/cm^2^ atm day permeable films	[167]
20	Active NanoPackaging with nano-zinc oxide in PVC	Fresh cut Fuji apples	Storage at 4 °C for 12 days	✓ Significant reduction in fruit decay rate ✓ MDA content reduced in 53.9 nmol/g as against control 74.9 nmol/g ✓ Decreased activity of PPO and POD ✓ Reduced browning index 23.9 as compared with control 31.7	[168]
21	Active packaging using Pinhão starch/citrus pectin/Feijoa Peel Flour (0, 0.4, 1, 2, 3 and 4% application)	Apples	Storage at room temperature for 15 days	✓ Exhibited a reduced weight loss during storage	[169]
22	Nano-packaging with PE	Strawberry	Storage at 4 °C for 12 days	✓ Reduced the loss of TSSs, ascorbic acid, and TA ✓ Reduced decay rate (26.8%), MDA content (66.3 μmol/g)	[170]
23	Edible coating with Gum Arabic	Apricots	Storage at 20 ± 1 °C for 8 days	✓ Lowered weight loss in coated fruits ✓ Reduction in the content of H_2_O_2_, MDA with 1.41X and 1.35X compared with control	[171]

## Data Availability

No new data were created or analyzed in this study.
